# Hypothetical Protein VDAG_07742 Is Required for *Verticillium dahliae* Pathogenicity in Potato

**DOI:** 10.3390/ijms24043630

**Published:** 2023-02-11

**Authors:** Dahui Wang, Shenglan Wen, Zhibo Zhao, Youhua Long, Rong Fan

**Affiliations:** College of Agriculture, Guizhou University, Guiyang 550025, China

**Keywords:** Verticillium wilt, proteomic sequencing, hypothetical protein, conidial penetration, early infection

## Abstract

*Verticillium dahliae* is a soil-borne pathogenic fungus that causes Verticillium wilt in host plants, a particularly serious problem in potato cultivation. Several pathogenicity-related proteins play important roles in the host infection process, hence, identifying such proteins, especially those with unknown functions, will surely aid in understanding the mechanism responsible for the pathogenesis of the fungus. Here, tandem mass tag (TMT) was used to quantitatively analyze the differentially expressed proteins in *V. dahliae* during the infection of the susceptible potato cultivar “Favorita”. Potato seedlings were infected with *V. dahliae* and incubated for 36 h, after which 181 proteins were found to be significantly upregulated. Gene ontology and Kyoto Encyclopedia of Genes and Genomes enrichment analyses showed that most of these proteins were involved in early growth and cell wall degradation. The hypothetical, secretory protein with an unknown function, VDAG_07742, was significantly upregulated during infection. The functional analysis with knockout and complementation mutants revealed that the associated gene was not involved in mycelial growth, conidial production, or germination; however, the penetration ability and pathogenicity of *VDAG_07742* deletion mutants were significantly reduced. Therefore, our results strongly indicate that VDAG_07742 is essential in the early stage of potato infection by *V. dahliae*.

## 1. Introduction

*Verticillium dahliae*, a hemibiotrophic pathogen, is a highly virulent and destructive phytopathogenic fungus that causes Verticillium wilt in more than 600 plant species worldwide, many of them crop species, leading to great economic losses [1,2,3]. The infectious cycle of *V. dahliae* begins with the germination of the microsclerotia to form infective hyphae, which adhere to the host root surface to form hyphopodia that penetrate the host plant root epidermal cells before continuing to invade and block vascular vessels, eventually causing vascular tissue discoloration, leaf chlorosis, and necrosis, and ultimately, plant death [4,5]. The pathogenic mechanism of this fungus is very complex, with the infection process being divided into three stages: (1) in the early stage, spores germinate to form hyphae and adhere to the plant roots; (2) in the intermediate stage, the hyphae successfully invade the root epidermal cells, causing fungal colonization; (3) at the late stage of infection, wilting symptoms appears in the aboveground plant body [6]. The initial course of infection varies among the hosts and even within the hosts, differing in terms of resistance [7,8].

Several proteins, including key pathogenesis-related proteins, have been identified as influencing the *V. dahliae* infection [9,10,11]. Further, many hypothetical proteins without functional annotation reportedly play critical roles in fungal infection and pathogenesis, thus, for example, the hypothetical protein MoOeIF3k can positively regulate the penetration and colonization of rice by *Magnaporthe oryzae*, whereas, its deficiency results in significantly reduced pathogenicity [12]. Similarly, the deletion of the hypothetical protein-encoding gene *Fosp9* of *Fusarium oxysporum* reportedly hinders the early infection of banana plants significantly [13].

The wide application of bio-sequencing technology has resulted in the identification of an increasing number of hypothetical fungal proteins due to their interaction with the host; however, the lack of functional information greatly limits our understanding of fungal pathogenic mechanisms. Particularly, the identification and functional analysis of potential key hypothetical proteins involved in *V. dahliae* infection require a thorough understanding of the mechanism responsible for the pathogenesis of the fungus.

The transcriptome analysis of *V. dahliae* during infection has provided new insights into the fungal infection mechanism, and many critical genes related to its pathogenesis have been identified; however, no simple correspondence between the transcript and protein expression has been observed [14]. In contrast, proteomics has been shown to be a useful tool for identifying the differentially expressed proteins involved in fungal infection [15]. Thus, for example, based on proteome analysis, the ester cyclase UvEC1 was found to be essential for the development and pathogenicity of *Ustilaginoidea virens* [16], and pectinases VdPL3.1 and VdPL3.3 were found to be upregulated in the extracellular proteome of *V. dahliae* in a cotton-containing medium [17]. Furthermore, a conserved, secreted VdCP1 protein was also identified by proteomics, and the deletion of the corresponding gene reduced the *V. dahliae* pathogenicity in cotton [18]. These results suggest that proteomics analysis can help identifying the key pathogenic proteins involved in *V. dahliae* infection.

Potato Verticillium wilt caused by *V. dahliae*, also known as potato early dying disease, is a serious problem for potato cultivation worldwide. However, only a little bit is known about the key pathogenesis-related proteins, especially those with unknown functions, involved in the initial infection stages of the fungus. Therefore, proteomic analysis was conducted in the present study to identify the potential key proteins responsible for the pathogenesis of *V. dahliae*, and a hypothetical protein VDAG_07742 was selected for further functional study by generation of the *VDAG_07742* deletion and complementary mutants, thus confirming its importance in the pathogenesis of Verticillium wilt in potatoes.

## 2. Results

### 2.1. Differentially Expressed Proteins (DEPs) Identified during V. dahliae Infection of Potato Plants

**Figure 1:**
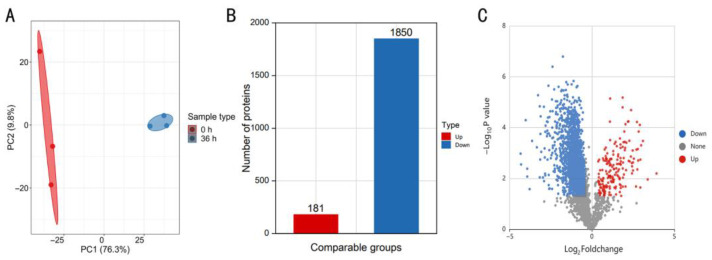
Overview of protein identification and sample repeatability test. (**A**) Principal component analysis (PCA) showed quantitative sample repeatability, (**B**) histogram of the protein number distribution of differentially expressed proteins (DEPs) in different comparison groups, and (**C**) volcano plot of DEPs in the groups being compared (|log_2_ Fold Change| > 1.3 and *p* < 0.05).

To gain new insights into DEPs during early infection of potatoes by *V. dahliae*, proteins induced at 0 and 36 h after the *V. dahliae* infection were subjected to proteomic analysis and sequenced using quantitative proteomics with tandem mass tag (TMT). Subsequently, a principal component analysis (PCA) was performed to assess the overall quality and comparability of the proteomic datasets obtained from *V. dahliae* conidia (0 h) and conidia-inoculated potato roots (36 h). The PCA plots generated from the normalized data representing the treatments and experimental replicates showed a clear difference on the PC1 axis at 0 and 36 h after inoculation (Figure 1A), indicating that the biological effects were stronger than the technical effects were; therefore, the quality of the data was considered to be sufficient for further analysis. A comparison between the proteomes observed at 0 and 36 h after inoculation indicated the presence of 2031 DEPs (Figure 1B), 181 of which were upregulated (Figure 1C). Details of the upregulated proteins are provided in Table S1. Among these, 23 proteins were upregulated more than five-fold (Table 1).

### 2.2. Functional Cluster Analysis of Upregulated Proteins

**Figure 2:**
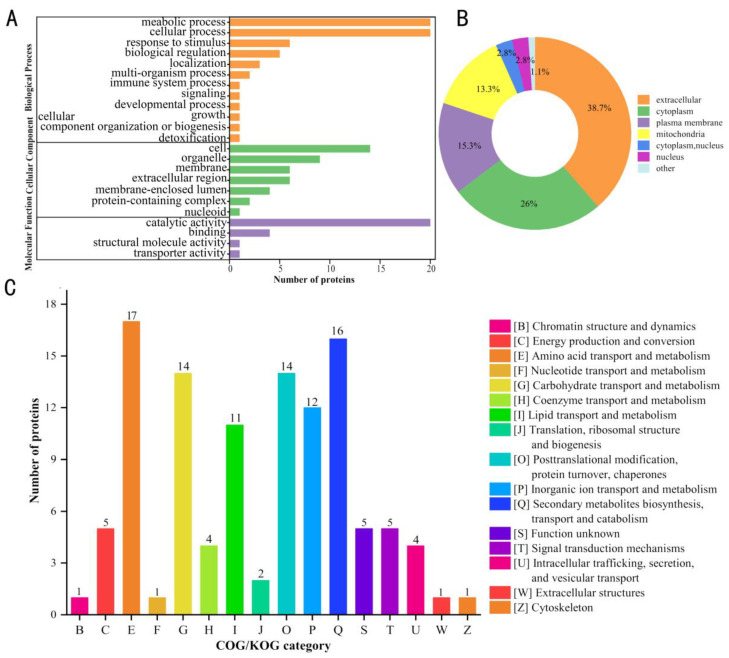
Classification of upregulated proteins. (**A**) Gene ontology (GO) functional classification, (**B**) subcellular locations, and (**C**) clusters of orthologous groups (COG) showing functional classes.

We hypothesized that some of the 181 upregulated proteins upon fungal infection may play important roles in *V. dahliae* pathogenesis. To further understand the functions of these proteins, gene ontology (GO) functional classification was performed using the GO database. The results show that the upregulated proteins were mainly involved in metabolic processes such as catalysis and binding (Figure 2A) and cellular processes. In contrast, the subcellular localization showed that the upregulated proteins were distributed within the extracellular space (70%), in the cytoplasm (47%), and in the plasma membrane (28%) (Figure 2B). A further analysis of the upregulated proteins observed via the clustering of orthologous groups (COG) suggested that they could be divided into 23 groups, with seventeen proteins in the largest group associated with amino acid transport and metabolism, and only one in the smallest group associated with processes such as cytoskeleton production, extracellular structures, nucleotide transport and metabolism, and chromatin structure and dynamics (Figure 2C and Table S2).

### 2.3. Functional Enrichment Analysis of Upregulated Proteins

**Figure 3:**
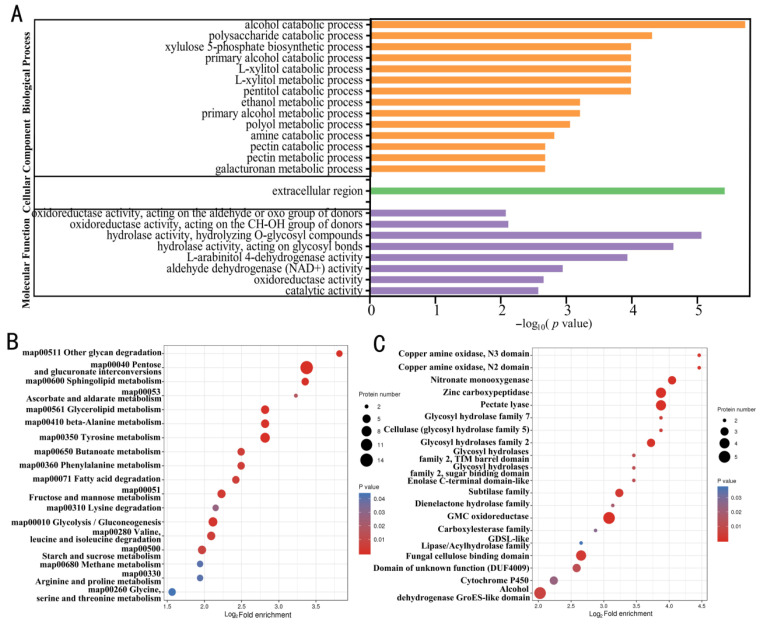
Enriched gene ontology (GO) terms, Kyoto encyclopedia of genes and genomes (KEGG) pathways, and protein domains of the proteins showing upregulated expression levels in the comparison groups. (**A**) Cellular components, molecular functions, and biological processes, (**B**) enriched KEGG pathways, and (**C**) enriched protein domains.

The GO enrichment analysis showed that upregulated proteins were mainly enriched in terms of alcohol decomposition, polysaccharide decomposition, primary alcohol decomposition, pentose alcohol decomposition, and L-xylitol metabolism. Furthermore, the cellular components were significantly enriched in the extracellular regions, and molecular function enrichment was observed mainly in hydrolase activity, hydrolytic O-glycosyl compounds, and oxidoreductase activity (Figure 3A). Consistently, the Kyoto Encyclopedia of Genes and Genomes (KEGG) metabolic pathway analysis showed that 100 upregulated proteins were involved in 18 pathways, among which, the most significantly main enriched ones were pentose and glucuronate interconversions (map00040), tyrosine metabolism (map00350), and glycolysis/gluconeogenesis (map00010) (Figure 3B, Table 2 and Table S3). The protein domain enrichment results showed that 67 upregulated proteins were enriched in different domains, of which the most enriched domains were GMC oxidoreductase, alcohol dehydrogenase GroES-like domain, and pectate lyase (Figure 3C, Table 3 and Table S4).

Based on the GO enrichment, KEGG enrichment, and domain enrichment analyses, information was obtained for 152 out of the 181 upregulated proteins. Notably, 29 hypothetical proteins with unknown functions were significantly upregulated during the early stages of *V. dahliae* infection (Table S5).

### 2.4. qRT-PCR Verification of Upregulated Proteins

**Figure 4:**
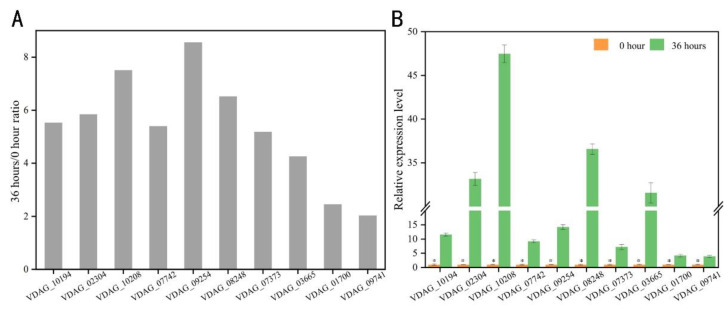
Real-time quantitative PCR (qRT-PCR) verification of proteins whose expression was upregulated at 0 and 36 h after *Verticillium dahliae* infection in potatoes. (**A**) Relative expression level ratios of 10 randomly selected proteins at 36/0 h in the proteomic data; (**B**) the relative expression was verified at the mRNA level using *β-tubulin* as a reference gene. Values on the vertical axis represent means ± standard error (SE) of three biological replicates. Asterisks represent significant differences relative to 0 h, as determined by the least significant difference (LSD) test at *p* = 0.05.

A quantification of the mRNA expression levels of the upregulated *V. dahliae* proteins in the infected potato plants at 0 and 36 h post-inoculation of conidia was performed by qRT-PCR, for which, the following 10 upregulated proteins were randomly selected: VDAG_10194, VDAG_02304, VDAG_10208, VDAG_07442, VDAG_09254, VDAG_08248, VDAG_07373, VDAG_03665, VDAG_01700, and VDAG_09741. Basic information describing these proteins and primers used for qRT-PCR verification are listed in Tables S1 and S6, respectively. Consistent with proteomic data obtained from TMT quantification, the mRNAs of all 10 proteins were more upregulated at 36 h compared to those at 0 h (Figure 4), demonstrating the reliability of the proteomic analysis.

### 2.5. Identification and Characterization of the VDAG_07742 Protein in V. dahliae

**Figure 5:**
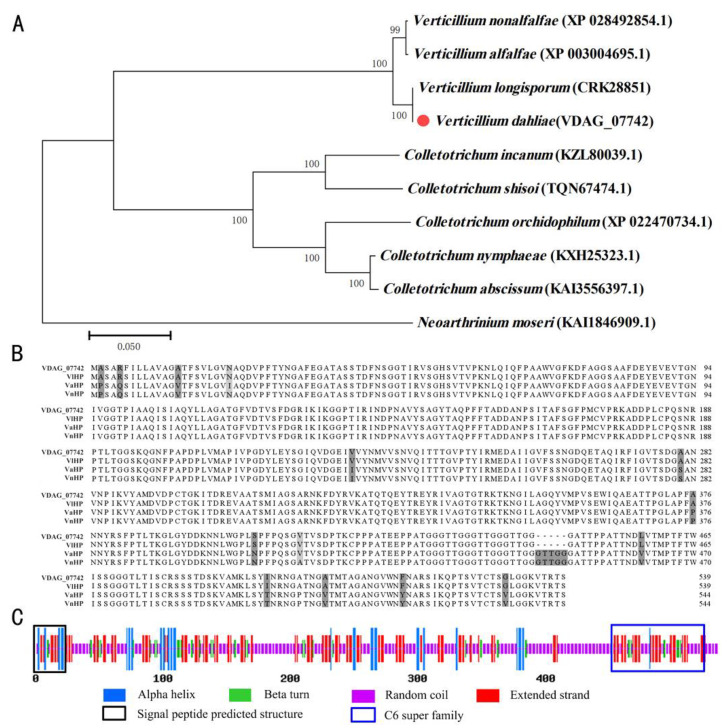
Characterization of VDAG_07742 in *Verticillium dahliae*. (**A**) Phylogenetic tree constructed using the MEGA7.0 neighbor-joining method using 9 fungal amino acids with bootstrap percentages obtained from 1000 replicates at each branch node and the red dot represented the amino acid sequence of VDAG_07742; (**B**) multiple sequence alignments showing homologous proteins in *V. nonalfalfae*, *V. alfalfae,* and *V. longisporum*; Non-conserved amino acid residues were shaded in gray and the darker the color, the less conserved the residue. (**C**) secondary structure prediction of VDAG_07742.

Based on the proteomic analysis, the expression of a hypothetical protein VDAG_07742 was upregulated as high as 5.4-fold. A BLASTP search for VDAG_07742 revealed 100% similarity with the hypothetical proteins that have been previously described in *V. nonalfalfae*, *V. alfalfae*, and *V. longisporum*. Protein sequences with high similarity were downloaded for the phylogenetic analysis, which showed that VDAG_07742 belongs to the same branch as the Verticillium fungi (Figure 5A). Indeed, the multiple sequence homology analysis showed that VDAG_07742 is highly conserved in Verticillium fungi (Figure 5B). We speculated that the protein may play important roles in the pathogen–host interaction in *Verticillium* sp., so the protein was selected for the functional analysis in this study.

The gene sequence of *VDAG_07742* was amplified using the genomic DNA of *V. dahliae* wild-type (WT) strain JY, and it was 100% identical with that of the *V. dahliae* VdLs.17 in the JGI (https://genome.jgi.doe.gov/ (accessed on 8 January 2023)) database. The VDAG_07742 protein with 530 amino acids, which is a non-GPI-anchored protein that contains a signal peptide at the position associated with fragment 1–23 and located in the extracellular space without a transmembrane domain, additionally, it contains a C6 super family domain at fragment 455–527 (Figure 5C). The predicted secondary structure of the protein contained an alpha helix (9.46%), a beta turn (7.98%), random coils (54.17%), and extended strands (28.39%; Figure 5A).

### 2.6. Vegetative Propagation, Conidial Production, and Spore Germination of Mutants

**Figure 6:**
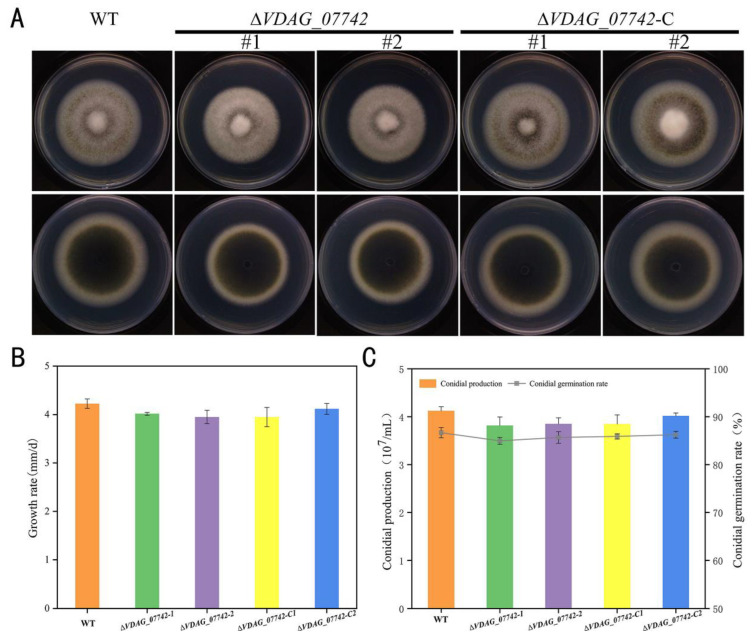
Analysis of fungal growth, conidial production, and conidial germination rate of *VDAG_07742* deletion mutants. (**A**) Colony morphology of wild-type (WT) *Verticillium dahliae* strain JY, knockout mutants *ΔVDAG_07742*-1 and *ΔVDAG_07742*-2 and complementary mutants *ΔVDAG_07742*-C1 and *ΔVDAG_07742*-C2 cultured on PDA plates for 10 d; (**B**) colony growth rate; (**C**) conidial production and conidial germination rate. Values on the vertical axis represent the mean ± standard error (SE) for three biological replicates. Asterisks represent significant differences relative to the WT, as determined by the least significant difference (LSD) test at *p* = 0.05.

To clarify whether *VDAG_07742* plays a role in *V. dahliae* vegetative growth, two *VDAG_07742* deletion mutants (Δ*VDAG_07742*-1 and Δ*VDAG_07742*-2) and two *VDAG_07742* complementary mutants (Δ*VDAG_07742*-C1 and Δ*VDAG_07742*-C2) were used for further study after the genetic transformation and PCR verification (Figure S1) [3,19,20]. Following cultivation on a PDA medium for 10 d, both of the knockout mutants showed the same colony morphology and color as those of the WT strain and complementary mutants (Figure 6A). Furthermore, no significant differences were observed between the mutants and WT in the colony growth rate, conidial production, or spore germination (Figure 6B,C).

### 2.7. Conserved Hypothetical Protein VDAG_07742 Is Required for V. dahliae Pathogenicity to Potato Plants

**Figure 7:**
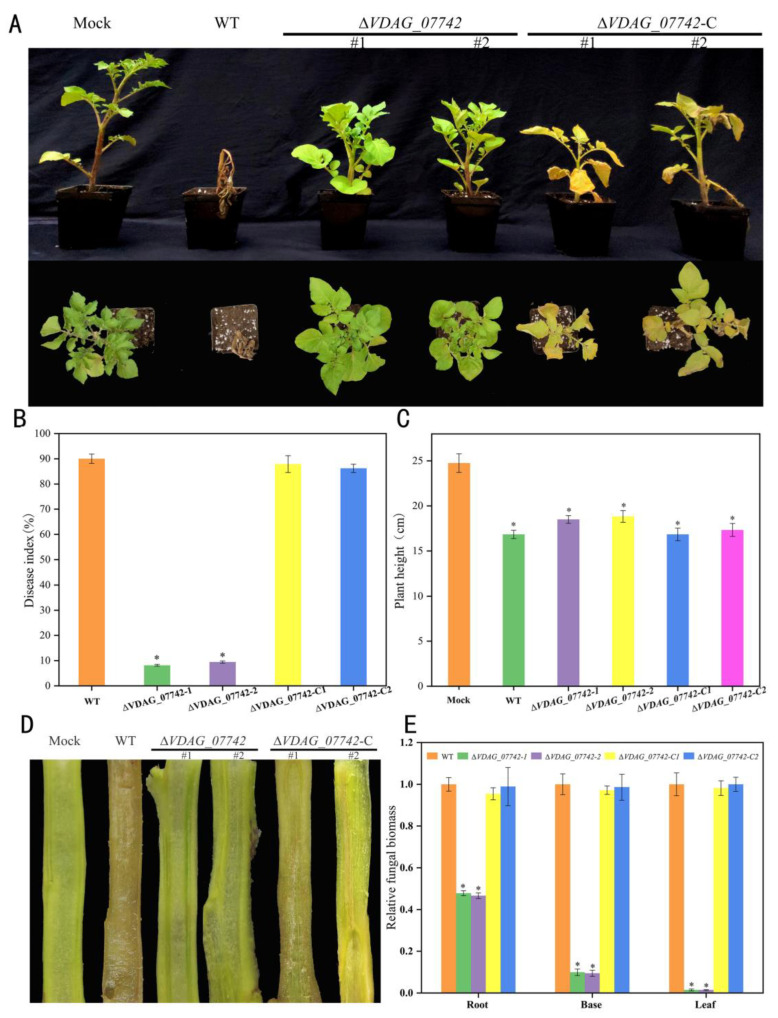
*VDAG_07742* is required for pathogenicity of *Verticillium dahliae*. (**A**) Potato seedlings at the 6-leaf stage were inoculated with spore suspensions of wild-type (WT) *V. dahliae* strain JY, the knockout mutants *ΔVDAG_07742*-1 and *ΔVDAG_07742*-2, and complementary mutants *ΔVDAG_07742*-C1 and *ΔVDAG_07742*-C2, for four weeks; (**B**) disease index showing Verticillium wilt; (**C**) potato plant height; (**D**) discoloration of vascular vessels in longitudinal potato plant slices; (**E**) *V. dahliae* biomass using potato *actin* as a reference gene. Values on the vertical axis represent the mean ± standard error (SE) of three biological replicates. Asterisks represent significant differences relative to WT (control), as determined by the least significant difference (LSD) test at *p* = 0.05.

To investigate the role of *VDAG_07742* on *V. dahliae* pathogenicity, *V. dahliae*-susceptible potato cultivar “Favorita” was used for inoculation [21]. The *V. dahliae* WT strain and mutants were inoculated into potato seedlings at the six-leaf stage using the dipping root method [22]. Four weeks after inoculation, the plants inoculated with the WT strain showed obviously wilt symptoms, whereas those inoculated with mutants or the mock ones did not, and the complementary mutants caused more severe chlorosis of typical wilt symptoms than the deletion mutants did (Figure 7A). The disease index results show that the deletion mutants caused a significantly less severe wilt disease (Figure 7B), and the heights of the potato plants infected with the *V. dahliae* strains were less than the mock plants were, but no difference was detected between these strains (Figure 7C). No discoloration was observed in the vascular vessels of the plants infected with the deletion mutants, which appeared to be similar to the mock plants. In contrast, discoloration was evident in the WT strain and the complementary mutants (Figure 7D). The qRT-PCR results further confirmed that the biomass of deletion mutants was significantly lower than those of the WT or complementary mutants in the root, base, or leaf tissues (Figure 7E), indicating the colonization of *V. dahliae* was defective without the gene *VDAG_07742*. In addition, the expression of *VDAG_07742* in the early infection process in the WT strain was significantly induced (Figure S2). Therefore, the results above indicate that *VDAG_07742* is required for the pathogenicity of *V. dahliae* in potatoes.

### 2.8. Hypothetical Protein VDAG_07742 Impairs the Penetration Ability of V. dahliae

**Figure 8:**
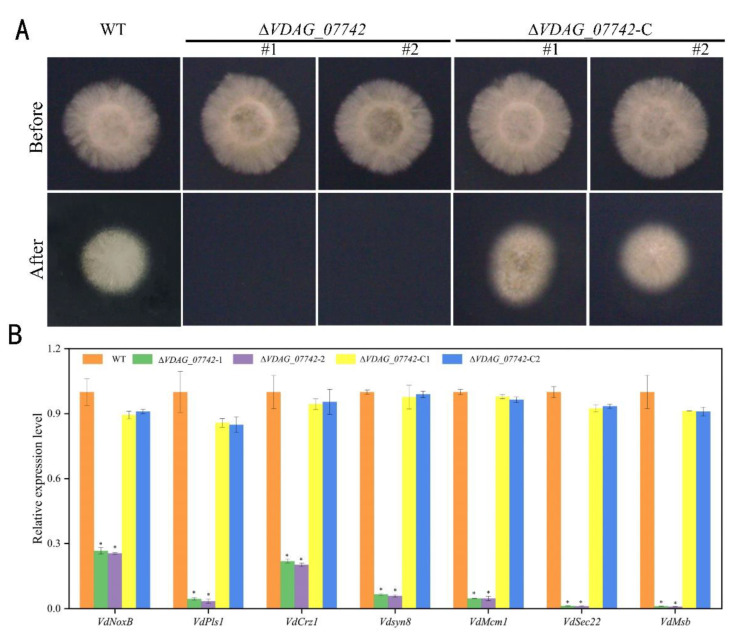
*VDAG 07742* influences *Verticillium dahliae* penetration. (**A**) The first row shows the growth of wild-type (WT) *V. dahliae* strain JY, knockout mutants *ΔVDAG_07742*-1 and *ΔVDAG_07742*-2, and complementary mutants *ΔVDAG_07742*-C1 and *ΔVDAG_07742*-C2 cultured on cellulose membrane-covered PDA plates for 3 d; the second row shows the growth of the WT strain, knockout mutants *ΔVDAG_07742*-1 and *ΔVDAG_07742*-2, and complementary mutants *ΔVDAG_07742*-C1 and *ΔVDAG_07742*-C2 5 d following removal of the cellulose membrane; (**B**) expression of infection-related *V. dahliae* genes detected by qRT-PCR using *β-tubulin* as the reference gene. Values on the vertical axis represent the mean ± standard error (SE) of three biological replicates. Asterisks represent significant differences relative to the WT, as determined by the least significant difference (LSD) test at *p* = 0.05.

As a defective colonization was observed in the deletion mutants, considering that the vegetative growth was not affected by *VDAG_07742* in *V. dahliae*, we hypothesized that the penetration of *VDAG_07742* deletion mutants could be deficient. In order to detect the penetration ability, WT and mutant conidia were inoculated on PDA plates coated with cellulose membranes following the previously described method [23]. The removal of the cellulose membranes 3 d after inoculation and further culturing for 5 d rendered the knockout mutants unable to penetrate the cellulose membranes in the same manner as they did in the WT strain, while the penetration ability of the complementary mutants was restored (Figure 8A), indicating that the deletion of *VDAG_07742* damaged the ability of *V. dahliae* to penetrate the cellulose membranes. Previous studies showed that *VdNoxB*, *VdPls1*, and *VdCrz1* are essential for *V. dahliae* penetration [22,24], and our qRT-PCR results show that the expression of these genes was significantly reduced in the knockout mutants (Figure 8B). Further, the expression levels of the attachment-related genes *VdMsb* and *VdMcm1* [25,26] and the secretion-related genes *VdSec22* and *VdSyn8* [27] were also downregulated in the knockout mutants (Figure 8B). Altogether, these results indicate that the penetration ability of the knockout mutants was hampered compared to that of the WT fungal strain, and this effect was due to a reduced expression of penetration-related genes, while the attachment and secretion related genes were positively regulated during infection.

## 3. Discussion

Several pathogenesis-related proteins are involved in *V. dahliae* infections, and many of them are hypothetical proteins with unknown functions, which greatly limits our understanding of the pathogenicity mechanisms of *V. dahliae*. Therefore, it is important to explore the pathogenicity-related proteins and clarify their functions to help to develop new strategies of Verticillium wilt. In this study, a proteomic analysis was used to identify the proteins involved in *V. dahliae* infection of potato, especially those with unknown functions. The results showed that 181 proteins were significantly upregulated expressed in early infection process, of which, 29 proteins have unknown functions. Particularly, an upregulated hypothetical protein VDAG_07742 was explored due to its high conservatism in *Verticillium* sp. Indeed, the bioinformatic analysis showed that VDAG_07742 is a typical secretory protein, and a functional assessment confirmed its importance for *V. dahliae* pathogenicity to potatoes in this study.

The proteomic data showed that 181 proteins were upregulated 36 h after infection relative to those at 0 h. The subsequent verification of the expression trend of 10 randomly selected proteins by qRT-PCR was consistent with the results obtained by the proteomic analysis, indicating the high accuracy of the proteome sequencing. Further, the GO classification of the biological processes in these upregulated proteins showed that most of them were related to cell and growth processes. In contrast, the COG classification indicated that the upregulated proteins were mainly involved in activities concerning the cytoskeleton, extracellular structures, posttranslational modification, protein turnover, chaperones action, translation, ribosomal structure, and biogenesis. Additionally, the KEGG enrichment analysis suggested that the upregulated proteins were mainly involved in tyrosine metabolism and glycolysis. Thus, all these proteins are closely related to the growth and development of *V. dahliae* [26,27,28,29,30,31,32], for which spore germination and invasive hyphal growth are often the initial steps of the infection. Our results provide evidence that cell proliferation-related proteins are actively expressed at the early stages of infection, which is consistent with the available bioinformatic results [23,32,33,34]. During the infection process, extracellular proteases are secreted to degrade the cell wall components of the host plant and obtain the nutrients needed for growth, moreover, the plant cell walls degraded in this manner promote *V. dahliae* infection [35,36]. Consistently, our GO enrichment analysis of the molecular functioning of the proteins under study showed two hydrolase activity entries involved in the hydrolysis of O-glycosyl compounds and glycosyl bond activity, which were significantly enriched. Consequently, these two entries were also significantly enriched in cotton plants with different resistances at the early stage of *V. dahliae* infection [17], and they also play a role in the interactions between other pathogenic fungi and host plants [37,38], indicating that the proteins in these two entries are highly conserved and closely related to the pathogenicity of the fungus. The two entries obtained in this study include a total of 12 proteins from the glycosyl hydrolase family, such as pectate lyase, and cellulase, with VDAG_07238, VDAG_07825, and VDAG_04977 reportedly being closely related to *V. dahliae* pathogenicity [17,39,40]. This study further demonstrated that proteomics research can be applied for the effective identification of the key pathogenic proteins involved in infection by *V. dahliae* or other pathogenic fungi. In addition, 29 other candidate proteins with unknown functions and roles in fungal pathogenicity have not been previously reported. A further study to elucidate their functions in the infection process might contribute to a thorough understanding of the pathogenic mechanism of *V. dahliae*.

Secreted proteins affect the colonization and pathogenicity of *V. dahliae* in host plants by changing the structure and function of the host cells to promote infection or control growth [41,42]. The bioinformatics analysis suggested that VDAG_07742 is a typical secreted protein that contains a C6 superfamily domain. However, to date, no unifying function has been described for the C6 superfamily domain. Therefore, the functional analysis of *VDAG_07742* in this study provides new insights into the regulatory role of the C6 superfamily domain in fungal infection. The vegetative growth of hyphae and the production of conidia during the infection and colonization of the host plants by *V. dahliae* can damage the vascular vessels and lead to cell necrosis and the discoloration of the vascular bundles [43,44].

In this study, the deletion of *VDAG_07742* significantly hindered *V. dahliae* colonization, with less vascular vessel discoloration being observed in the inoculated plants (Figure 7), while it had no effect on fungal mycelial growth, conidial production, or spore germination (Figure 6). Therefore, we hypothesize that the reduced extent of colonization and limited pathogenicity in Δ*VDAG_07742* mutants most likely result from a reduced ability of the fungus to penetrate the host rather than from the reduction of the vegetative growth. Indeed, further experiments showed the defective penetration of a cellulose membrane by the *VDAG_07742* knockout mutants and limited fungal growth by a reduced ability of the mutant *V. dahliae* to penetrate the root cell wall. Previous studies showed that the penetration ability of *V. dahliae* is closely related to its pathogenicity [5,45,46]. Specifically, *VdNoxB* and *VdPls1* are reportedly related to the formation of infection of the nails during *V. dahliae* penetration [24] and the deletion of the NADPH oxidase A-encoding gene *NoxA*, significantly and concomitantly reduced the pathogenicity and the ability of *V. dahliae* to penetrate cellophane [47]. Similarly, *VdSho1*, which encodes a tetraspanin, is required for *V. dahliae* penetration into cotton plants, and a deletion mutant showed only the slight discoloration of the vascular vessels, concomitant with a significant reduction of pathogenicity [23]. Consistently, the qRT-PCR results reported to date also show that the genes *VdSec22* and *VdSyn8,* which are related to secretion [5,27], *VdMsb* and *VdMcm1,* which are associated with attachment [25,26], and *VdCrz1*, which is involved in signal transduction [22], are all significantly downregulated in the *VDAG_07742* deletion mutants, further confirming the involvement of VDAG_07742 in *V. dahliae* pathogenicity.

The infection mechanism of *V. dahliae* is complex. Numerous potential proteins are involved in the pathogenic process; however, the functions of these proteins remain unknown. Determining the potential key proteins in the infection process of *V. dahliae* and clarifying their functions are important for comprehensively elucidating the pathogenic mechanism of *V. dahliae* and exploring novel plant disease control approaches. In this study, VDAG_07742, a hypothetical protein involved in the early stage of the infection process of potatoes by *V. dahliae*, was identified through proteomic analysis, and its role in pathogenicity was clarified, thus providing novel insights into the pathogenic mechanism of *V. dahliae* in potato plants.

## 4. Materials and Methods

### 4.1. Plant Material and Strains

Susceptible potato (*Solanum tuberosum* L) cultivar “Favorita” was used for the experiments. Potato seedlings were planted in sterilized soil under a 28/25 °C (day/night), temperature regime, and an 8/16 h (day/night) photoperiod [21]. Virulent *V. dahliae* strain JY was isolated from Shaanxi Province, China, and cultured in PDA (potato 200 g/L, glucose 20 g/L, agar 15 g/L) medium at 25 °C. [48]. The knockout plasmid pA-Hyg-OSCAR and overexpression vector pFL2 were donated by Professor Hu Xiaoping at the College of Plant Protection, Northwest Agriculture and Forestry University, Xianyang City, Shaanxi Province, China. *Escherichia coli*-competent DH5α was purchased from TransGen Biotech (Beijing, China).

### 4.2. V. dahliae Inoculation and Sample Preparation

Healthy potato seedlings at the six-leaf stage were selected as inoculum subjects. *V. dahliae* JY strain was cultured in PDA and sterile water for 7 d to collect conidia. Then, the concentration of the conidia suspension was adjusted to 10^7^ conidia/mL using a blood count plate. Inoculum culture was performed according to previously describe d methods [3]. Potato plants inoculated with conidia for 0 h were used as controls, and experimental plants were incubated at 25 °C/80 rpm for 36 h. Then, they were removed, the root surface mycelia were gently washed with sterile water, and samples were collected and immediately frozen in liquid nitrogen and stored at −80 °C until further use.

### 4.3. Protein Extraction, Digestion, and LC-MS/MS Analysis

Protein extraction, digestion, and LC-MS/MS analysis were performed according to previously described methods [15,16]. Briefly, the proteins were extracted in a lysis buffer (10 mM dithiothreitol, 1% protease inhibitor cocktail), pelleted, treated with acetone, and dissolved in 8 M urea. The protein concentration was determined using a BCA Kit (Beyotime Biotechnology, Shanghai, China) according to the manufacturer’s instructions. For digestion, the protein lysate was added with trichloroacetic acid (TCA) to obtain a final concentration of 20%, and left to precipitate for 2 h, after which, tetraethylammonium bromide (TEAB) was added to obtain a final concentration of 200 mM. Subsequently, trypsin was then added at a ratio of 1:50 (protease:protein, m/m), and the mixture was hydrolyzed overnight. Finally, dithiothreitol (DTT) was added to a final concentration of 5 mM, and the mixture was incubated at 56 °C for 30 min. Then, 3-indoleacetic acid (IAA) was added to achieve a final concentration of 11 mM, and the mixture was incubated at 25 °C for 15 min in the dark. After trypsin digestion, the peptides were dissolved via liquid chromatography with solvent A (0.1% formic acid and 2% acetonitrile) and separated using an EASY-nLC 1200 UHPL system (Thermo Fisher Scientific, Waltham, Massachusetts, USA). The data were analyzed using an Exploris 480 mass spectrometer (MS/MS). The peptide parent ions and their secondary fragments were detected and analyzed using Orbitrap, which was followed by a secondary MS/MS analysis. The MS/MS data were processed using PD2.4. SEQUST was then used along with the proteome database of *V. dahliae* VdLs.17 downloaded from UniProt. Tandem mass spectra were searched using the *V*. *dahliae* database, and a back library was added to calculate the false positive rate resulting from random matches (FDR). Trypsin/P was specified as the cleavage enzyme, and the TMT-6plex method was used to obtain quantitative values from the replicated 0 and 36 h samples. The PCA was performed to test the statistical consistency of the quantitative results. The average repeated quantitative value was determined for each sample, and the two-sample two-tailed *t*-test was used to calculate the *p*-value to determine the significance of the differential expression of proteins in the two samples. The DEPs with a |log_2_ Fold Change| > 1.3 compared to those in the 0 h sample were selected, and the differences at *p* < 0.05 were considered to be significant.

### 4.4. Functional Annotation and Enrichment Analysis

For the functional classification, the upregulated proteins were annotated according to the GO terms based on the *V. dahliae* VdLs.17 database and subjected to subcellular localization and COG functional classification analysis. The selected significant terms were used to generate graphical results to simplify and outline the data. Subsequently, all the GO annotation information was used as a background file for the GO enrichment, KEGG enrichment, and protein domain enrichment analysis. The GO terms within the upregulated protein sets with corrected *p* values of <0.05 and upregulated proteins at *p* < 0.05 significance level as per the two-tailed Fisher’s exact test were considered to be significantly enriched.

### 4.5. qRT-PCR Analysis

To evaluate how well the gene expression levels matched our quantitative proteomics data, the qRT-PCR analysis was performed using 10 upregulated proteins that were randomly selected. Primer 5.0 was used to design primers for the genes encoding the 10 selected upregulated proteins and for the reference gene *β-tubulin*. First, RNA was extracted from the root samples of *V. dahliae*-infected potatoes plants at 0 and 36 h after inoculation and reverse-transcribed according to a previously described method [3]. qRT-PCR was performed using 2×RealStar Green Fast Mixture (20 µL volume) using a 10 μL 2× RealStar Fast SYBR qPCR Mix, 0.4 μL High/Low ROX Reference Dye, 7.6 µL ddH_2_O, 1 µL forward and reverse primers, and 1 µL cDNA. The steps for the qRT-PCR steps included 95 °C denaturation for 2 min, 40 cycles at 95 °C for 15 s, 60 °C for 30 s, and 72 °C for 30 s, and a melting curve. Biological replicates were performed three times, and the average values were calculated. *β-tubulin* was used as an internal reference gene to detect changes in the expression of the different treatment groups. Data were analyzed using the 2^−ΔΔCt^ method. The relative expression at 0 h was used for normalization [49].

### 4.6. Bioinformatics Analysis

The nucleic acid sequences for *VDAG_07742* were obtained from the *V. dahliae* database in JGI (https://genome.jgi.doe.gov/ (accessed on 8 January 2023)), and primers were designed to amplify the full length of the gene using the DNA of the JY fungal strain as a template before sequencing. The signal peptide, transmembrane structure, subcellular localization, and the GPI-anchored proteins were used for the VDAG_07742 amino acid sequence using SignalP4.1 (https://services.healthtech.dtu.dk/ (accessed on 7 November 2022)), TMHMM2.0 (https://services.healthtech. dtu. dk/service. php?TMHMM-2. 0 (accessed on 15 November 2022)), Cell-PLoc 2.0 (http://www.csbio.sjtu.edu.cn/bioinf/Cell-PLoc-2/ (accessed on 1 January 2023)), and PredGPI (http://gpcr.biocomp.unibo.it/predgpi/pred.htm (accessed on 15 November 2022)). The online software SOPMA (https://npsa-prabi.ibcp.fr/cgi-bin/npsa_automat.pl?page=npsa_sopma.html (accessed on 7 November 2022)) was used to predict the secondary structure of VDAG_07742. In contrast, amino acid sequence alignment was performed using BLASTP, the homologous sequences were downloaded, and a phylogenetic tree was constructed using MEGA7.0. Sequences with >99% homology were used for the multiple sequence alignment.

### 4.7. Generation of Knockout and Complementation Mutants, and Determination of Fungal Morphology

To determine whether the upregulated protein VDAG_07742 was involved in *V. dahliae* pathogenicity, gene-knockout of *VDAG_07742* was performedand pathogenicity assays were conducted. Targeted gene deletion plasmids were generated using a previously described method [3,19,20,50]. *VDAG_07742* deletion mutants were confirmed by PCR using the primers *hph*-F/R and Q-F/R, and complementary mutants were confirmed by the primers *neo*-F/R and Q-F/R. The primer sequences used are listed in Table S6.

The WT fungal strain, and deletion and complementary mutants were inoculated on PDA plates and cultured at 25 °C. The colony diameter was measured after 3, 6, 9, 12, and 15 days of being cultured to calculate the colony growth rate and observe the colony morphology. Conidia from the WT strain and from the deletion and complementary mutants were collected, and their concentrations were adjusted to 10^7^ conidia/mL, and 200 μL was aspirated and added to 50 mL PDB medium, followed by incubation at 25 °C/150 rpm for 7 d before the yield was determined using a blood cell counting plate. The concentration of the collected conidia was adjusted to 10^3^ conidia/mL, and 50 μL of each sample was dropped onto a sterile slide. After a 16 h incubation period at 25 °C, the germination rate of the conidia was determined. Three biological replicates were included in each experiment.

### 4.8. Pathogenicity Assays

The WT fungal strain JY and the deletion complementary mutants were cultured in liquid PDB medium for 7 d. Conidia were harvested by filtration through two layers of sterile filter cloth, and their concentration was adjusted to 10^6^ conidia/mL using sterile water. Uniformly grown healthy potato seedlings at the 6-leaf stage were used for the pathogenicity test. Briefly, the plants were inoculated using the root-dip method, with minor modifications [22]. Subsequently, the potato seedling roots were dipped in conidial suspensions for 10 min, and the seedlings were replanted into autoclaved sterile soil. The control seedlings were treated in the same way, but with sterile water instead of the conidia suspension. The potato seedlings were placed in a greenhouse at 25 °C and observed for four weeks. Twelve seedlings were used per treatment, and the pathogenicity assays were repeated at least three times. The seedling height was measured before the seedlings were removed from the soil and washed under running water. The degree of vascular tissue discoloration on the longitudinally cut stem was recorded and visually estimated, and disease severity was recorded on a scale from 0 to 5, where 0 = healthy leaves, 1 = plants showing slight chlorosis with <1% necrotic leaves, 2 = 40% of the plants showing chlorosis with 1–20% necrotic leaves, 3 = plants showing chlorosis with 20–30% necrotic leaves, 4 = plants showing chlorosis with 35–70% necrotic leaves, and 5 = plants showing chlorosis with 70–100% necrotic leaves. The percent disease index (DI) was determined using the standard formula: $\mathrm{DI}=\frac{\sum \left(\mathrm{Ni}\times \mathrm{Di}\right)}{N\times \mathrm{Dm}}\times 100$, where Di is the representative value for each level, Ni is the number of diseased leaves at each level, N is the total number of leaves investigated, and Dm is a representative value [23]. The plant height (PH) was measured from the base of the stem to the tip of the plant and calculated using to the formula: $\mathrm{PH}=\frac{\sum \left(\mathrm{Hi}\right)}{N}$, where Hi is the representative individual plant height, and N represents the total number of plant height measurements. Following a previous method, DNA was extracted from the potato roots, stems, and leaves infected with the WT strain and knockout and complementary mutants. *Actin* was used as the endogenous plant reference gene, and *V. dahliae* elongation factor 1-*α* (*VdEF-1α*) was used for the *V. dahliae* biomass quantification [3]. The primers used are listed in Table S6 in the Supplementary Information.

### 4.9. Penetration Assays

Conidia of each strain were collected, and their concentration was adjusted to 10^7^ conidia/mL. Sterile cellophane membranes were placed on a plate containing PDA medium as described previously, and equal amounts of conidia from each strain were placed in the center of the cellophane membranes [23]. The samples were then sealed and incubated at 25 °C for three days, after which, the cellophane membranes were removed from the plates, and these were further incubated for five days to determine the conidia penetration of the cellophane membranes. The transcription levels of infection-related genes in the WT and mutant strains were detected using qRT-PCR. Three biological replicates were included in each experiment. The tested genes and primers are listed in Table S6.

### 4.10. Statistical Analysis

All the experiments were conducted using three biological replicates, and the statistical analyses were performed using SPSS (version 16.0). Data were analyzed using one-way ANOVA and Duncan’s multiple range test. The treatment effects and differences were considered to be significant at *p* = 0.05. Origin 2021 and GraphPad Prism 8.30 were used for mapping and analysis, and Adobe Photoshop 2020 was used for image processing and output utilization. The GO analysis was performed using PANTHER (http://www.pantherdb.org/ (accessed on 20 October 2022)), and the KEGG analysis was performed using DAVID (https://david.ncifcrf.gov/ (accessed on1 November 2022)).

## 5. Conclusions

TMT-based quantitative proteomic analysis revealed that 181 proteins were significantly upregulated in *V. dahliae* at 36 h after the inoculation of conidia. Most of these proteins were associated with growth and cell wall degradation. Further, the functional analysis showed that the significantly upregulated hypothetical protein-encoding gene *VDAG_07742* is necessary for the host penetration and pathogenicity of *V. dahliae*. These results provide new insights into the mechanism responsible for *V. dahliae* pathogenicity in potato plants.

## Figures and Tables

**Table 1:** Proteins whose expression was upregulated by more than 5-fold in the proteomic data.

Protein ID ^a^	Protein Description	Fold Change ^b^	*p*-Value
VDAG_00499	Endothiapepsin	15.131	0.00639017
VDAG_07349	Cysteine-rich-protein	10.432	0.0109871
VDAG_09254	Glutathione-independent formaldehyde dehydrogenase	8.55	0.00033739
VDAG_10467	Dicarboxylic amino acid permease	7.929	0.00050033
VDAG_08662	4-coumarate-CoA ligase	7.667	0.00182525
VDAG_05550	General alpha-glucoside permease	7.659	0.02286991
VDAG_10208	Amino-acid permease inda1	7.505	7.8584 × 10^−5^
VDAG_07191	High-affinity nicotinic acid transporter	7.135	0.00014381
VDAG_07980	Aminopeptidase Y	7.004	0.0019787
VDAG_05967	Alkaline proteinase	6.903	0.00070141
VDAG_01172	4-coumarate:coenzyme a ligase(predicted)	6.76	0.0044525
VDAG_05650	Trypsin	6.648	6.0437 × 10^−5^
VDAG_08248	Hce2 domain-containing protein	6.522	0.00073655
VDAG_05344	Pectate lyase B	6.385	0.02084166
VDAG_02304	Aminopeptidase Y	5.838	0.00167567
VDAG_01113	EFG_II domain-containing protein(predicted)	5.636	0.00489088
VDAG_10194	Sodium/nucleoside cotransporter	5.626	0.00236321
VDAG_10460	Amino-acid permease inda1	5.549	0.00079813
VDAG_05115	Zinc carboxypeptidase A	5.42	0.00334736
VDAG_07742	Podospora anserina S mat+ genomic DNA chromosome 6, supercontig 4 (predicted)	5.4	0.00634638
VDAG_03907	Peptide hydrolase	5.35	0.00250297
VDAG_07373	Serin endopeptidase	5.188	2.0559 × 10^−5^
VDAG_07392	Cutinase (predicted)	5.154	0.00138373

^a^: Protein ID from *Verticillium dahliae* uniprot database. ^b^: Fold change value calculated by dividing the ratios of the tandem mass tag (TMT) reporter protein expression levels from 36 h to 0 h of infection with *V. dahliae.*

**Table 2:** List of the proteins whose expression was upregulated in the Kyoto encyclopedia of genes and genomes (KEGG) pathways.

Map	KEGG Pathway	Protein ID ^a^	Fold Enrichment	*p*-Value
Map00040	Pentose and glucuronate interconversions	VDAG_10081 VDAG_02904 VDAG_04977 VDAG_05799 VDAG_08495 VDAG_09741 VDAG_06080 VDAG_06523 VDAG_06079 VDAG_02886 VDAG_08496 VDAG_09536 VDAG_08929 VDAG_05344	10.37	11.3
Map00350	Tyrosine metabolism	VDAG_04798 VDAG_03345 VDAG_07316 VDAG_07507 VDAG_07314 VDAG_07369 VDAG_06372	7.04	4.49
Map00600	Sphingolipid metabolism	VDAG_05015 VDAG_05347 VDAG_01556 VDAG_07821	10.23	3.41
Map00561	Glycerolipid metabolism	VDAG_09648 VDAG_04951 VDAG_09741 VDAG_02013 VDAG_07738	7.04	3.31
Map00010	Glycolysis/Gluconeogenesis	VDAG_07446 VDAG_07507 VDAG_09741 VDAG_02013 VDAG_07057 VDAG_06372	4.33	2.69

^a^: Protein ID from *Verticillium dahliae* uniprot database.

**Table 3:** List of the proteins whose expression was upregulated in protein domain.

Domain Description	Protein ID ^a^	Fold Enrichment	*p*-Value
Zinc carboxypeptidase	VDAG_03588 VDAG_05115 VDAG_07183 VDAG_07290	14.67	4.25
Pectate lyase	VDAG_07238 VDAG_02904 VDAG_02886 VDAG_05344	14.67	4.25
GMC oxidoreductase	VDAG_00618 VDAG_08340 VDAG_05780 VDAG_07266 VDAG_05396	8.46	3.77
Nitronate monooxygenase	VDAG_01628 VDAG_07292 VDAG_00590	16.5	3.45
Glycosyl hydrolases family 2	VDAG_05015 VDAG_05347 VDAG_05015 VDAG_05347	14.67	4.25
Copper amine oxidase, N3 domain	VDAG_07314 VDAG_07369	22	2.69

^a^: Protein ID from *Verticillium dahliae* uniprot database.

## Data Availability

Not applicable.

## Supplementary Materials

The following supporting information can be downloaded at: https://www.mdpi.com/article/10.3390/ijms24043630/s1.
