# Supplementary material for: Hypothetical Protein VDAG_07742 Is Required for Verticillium dahliae Pathogenicity in Potato
Source: Int J Mol Sci. 2023 Feb 11;24(4):3630. doi: 10.3390/ijms24043630 (PMC9965449; doi:10.3390/ijms24043630)
Supplement: Supplementary file 1 [file ijms-24-03630-s001.zip › Figure S1.pdf]

International Journal of *Molecular Sciences*

MDPI

Article

Hypothetical Protein VDAG_07742 Is Required for *Verticillium dahliae* Pathogenicity in Potato

Dahui Wang, Shenglan Wen, Zhibo Zhao, Youhua Long and Rong Fan *

College of Agriculture, Guizhou University, Guiyang 550025, China
* Correspondence: rfan@gzu.edu.cn

This file includes Figure S1

To clarify the effect of VDAG_07742 on *Verticillium dahliae*, *VDAG_07742* was knocked out and complemented, and the correct knockout and complementation mutants were obtained according to the methods described in literature [1, 2]. Results showed that five mutants canbe detected using the primers of *hph*-F/R and Q-F/R (Table S6) targeting for hygromycin resistant marker on the plasmid and *VDAG_07742* on the genome of *V. dahliae* mutants, respectively (Figure S1A, S1B). Same as above, complementary mutants were confirmed using *neo*-F/R and Q-F/R primer pairs (Table S6) and named Δ*VDAG_07742*-C1 and Δ*VDAG_07742*-C2 (Figure S1C, S1D).

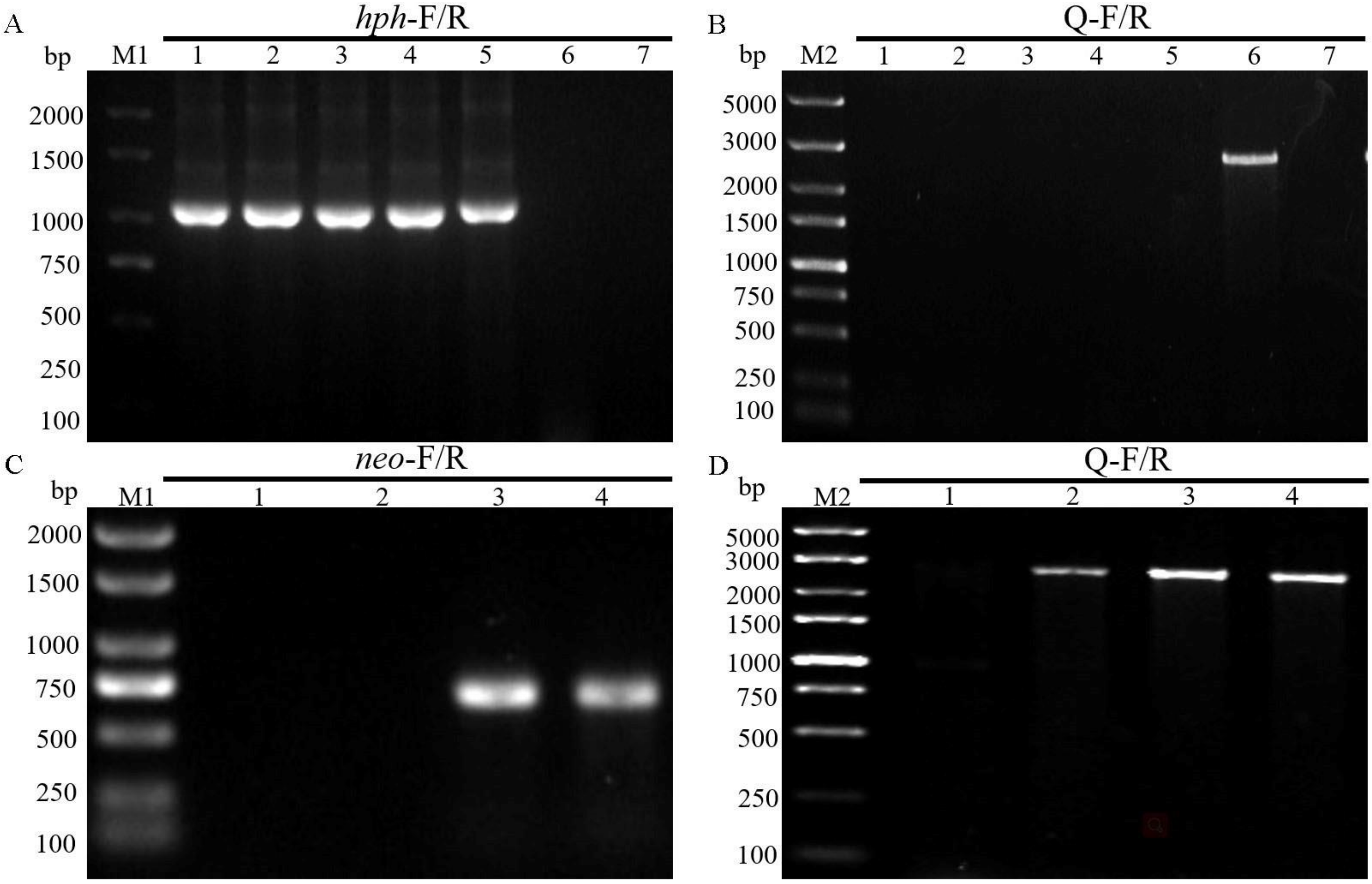

Figure S1. PCR confirmation of *VDAG_07742* knockout and complementation mutants. PCR detection of the knockout mutants using the primers of hph-F/R (A) and the target gene primers of Q-F/R (B). Lanes 1-5 represented DNA of five deletion transformants and lane 6 was the DNA of wild-type strain with the lane 7 of the negative control (using sterile

distilled water as the PCR template); PCR detection of the complementary mutants using the primers of neo-F/R (C) and the target gene primers of Q-F/R (D). Lane 1, the negative control; Lane 2 was the DNA of the wild type strain and lanes 3-4 were the DNA of two complementary transformants. M1, 2,000 bp marker; M2, 5,000 bp marker.

References

[1] Fan, R; Klosterman, S.J.; Wang, C.h.; Subbarao, K.V.; Xu, X.M.; Shang, W.J.; Hu, X.P. *Vayg1* is required for microsclerotium formation and melanin production in *Verticillium dahliae*. *Fungal Genet. Biol.* **2017**, *98,* 1-11. [CrossRef] [PubMed]

[2] Liu, T.; Qin, J.; Cao, Y.H.; Subbarao, K.V.; Chen, J.Y.; Mandal, M.K.; Xu, X.M.; Shang, W.J.; Hu, X.P. Transcription factor VdCf2 regulates growth, pathogenicity, and the expression of a putative secondary metabolism gene cluster in *Verticillium dahliae*. *Applied and environmental microbiology* **2022**. [CrossRef] [PubMed]
