# Supplementary material for: Hypothetical Protein VDAG_07742 Is Required for Verticillium dahliae Pathogenicity in Potato
Source: Int J Mol Sci. 2023 Feb 11;24(4):3630. doi: 10.3390/ijms24043630 (PMC9965449; doi:10.3390/ijms24043630)
Supplement: Supplementary file 1 [file ijms-24-03630-s001.zip › Figure S2.pdf]

International Journal of
Molecular Sciences

Article

Hypothetical Protein VDAG_07742 Is Required for *Verticillium dahliae* Pathogenicity in Potato

Dahui Wang, Shenglan Wen, Zhibo Zhao, Youhua Long and Rong Fan *

College of Agriculture, Guizhou University, Guiyang 550025, China
* Correspondence: rfan@gzu.edu.cn

This file includes Figure S2

In order to clarify whether *VDAG_07742* is involved in the early infection, qRT-PCR was used to detect the expression level of *VDAG_07742* in the wild type strain at 0 hpi, 24 hpi and 36 hpi. Results showed that the expression of *VDAG_07742* was significantly induced during the interaction of *V. dahliae* wild type strain with potato plants at 24 and 36 h post inoculation (Figure S2).

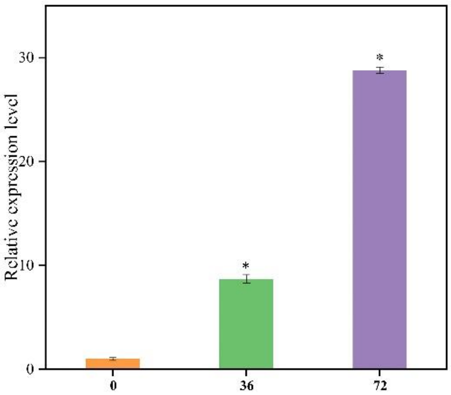

Figure S2. *VDAG_07742* expression in spores suspended in distilled water (0 h) and during the interaction of *V. dahliae* wild type strain with potato plants at 24 and 36 h post inoculation. Values on the vertical axis represent the mean ± standard error (SE) of three biological replicates. Asterisks represent significant differences relative to 0 h, as determined by the least significant difference (LSD) at $p = 0.05$
